# Trends in the effect of COVID-19 on consultations for persons with clinical and subclinical eating disorders

**DOI:** 10.1186/s13030-023-00285-2

**Published:** 2023-08-09

**Authors:** Keisuke Kawai, Hisateru Tachimori, Yurie Yamamoto, Yuki Nakatani, Shinmi Iwasaki, Atsushi Sekiguchi, Yoshiharu Kim, Naho Tamura

**Affiliations:** 1 Department of Psychosomatic Medicine Kohnodai Hospital, National Center for Global Health Medicine, 1-7-1, Kohnodai, Ichikawa City, 272-8516 Chiba Japan; 2 Chiba Prefecture Support Center for Eating Disorders, Ichikawa, Chiba Japan; 3 Endowed Course for Health System Innovation, Keio University School of Medicine, Tokyo, Japan; 4 Department of Clinical Epidemiology, Translational Medical Center, National Center of Neurology and Psychiatry, Tokyo, Japan; 5 Department of Pharmacy, Kohnodai Hospital, National Center for Global Health Medicine, Ichikawa, Chiba Japan; 6 National Center of Neurology and Psychiatry, Institute of Mental Health, Tokyo, Japan

**Keywords:** Eating disorders, Anorexia, Bulimia, COVID-19, Subclinical cases, School closure

## Abstract

**Background:**

The coronavirus disease 2019 (COVID-19) pandemic has increased the risk of individuals developing eating disorders and has exacerbated existing eating disorders. This observational study investigated the impact of the COVID-19 pandemic on patients with clinical and subclinical eating disorders.

**Methods:**

This study was conducted over a period of four years: two years before and after the onset of the COVID-19 pandemic in Japan. We recorded the number and types of consultations provided by the Eating Disorder Treatment and Support Center coordinator. For subgroup analysis, data were classified by age, body mass index, and source of consultation, including patients, families, and personnel. The Seasonal Decomposition of Time Series by Loess was used for time series analysis.

**Results:**

The total number of consultations increased after the start of the pandemic and peaked around the beginning of 2022, before subsequently falling despite the increase in the number of COVID-19 infections. A similar trend was observed in patients aged 10–29 years. The study period coincided with social isolation and school/college/university closures.

**Conclusions:**

The number of eating disorder consultations increased after the start of the pandemic. Although COVID-19 infections persisted, the pandemic’s impact was transient.

## Background

The coronavirus disease 2019 (COVID-19) is a respiratory infection caused by a new type of coronavirus called severe acute respiratory syndrome coronavirus 2 (or SARS-CoV-2) [1]. China first reported it to the World Health Organization in December 2019, and it has since proliferated worldwide. Globally, the cumulative number of infections and deaths reported was more than 496 million and 6 million, respectively, as of April 10, 2022 [2].

The global pandemic has affected people’s mental state. Specifically, the restrictions imposed in response to the COVID-19 pandemic have increased the risk of developing or worsening eating disorders (EDs) due to disrupted eating and exercise habits, social isolation, lack of support, and limited access to healthcare [3, 4]. One study found that the self-perceived mental health impact of COVID-19 changed over time but remained at concerningly high levels even one year after the pandemic’s onset, particularly for symptoms of depression and anxiety [5]. However, to our knowledge, no study has examined the variation in responses regarding EDs over time, before and after the COVID-19 pandemic, especially among the subclinical cases of individuals who have not visited a hospital and patients who have discontinued their visits and/or are considering a change in clinic. In a Finnish birth cohort study, the lifetime prevalence of anorexia nervosa was 2.2%, with several patients not visiting the hospital of their own volition and half of the cases remaining undetected in the healthcare system [6]. EDs are difficult to treat because of the associated stigma, patient denial of the severity of the illness, low motivation to change, negative attitudes associated with seeking help, and a lack of knowledge about relevant and helpful resources [7, 8]. As of 2015, the Japanese government had established four ED treatment support centers (Tohoku University Hospital, Miyagi Prefecture; Kohnodai Hospital of the National Center for Global Health and Medicine, Chiba Prefecture; Hamamatsu University Hospital, Shizuoka Prefecture; and Kyushu University Hospital, Fukuoka Prefecture). This study used data from the Chiba ED treatment support center. Two previous studies highlighted an increased prevalence of patients with EDs after the COVID-19 pandemic in Japan [9, 10]. However, both studies divided the study period into two broad categories: before and after the pandemic. Moreover, they both comprised a small sample size. The current study is the first to include more than 2,400 patients and analyze the subclinical ED cases of individuals who had never visited a hospital, using data collected both before and after the start of the COVID-19 pandemic.

This study was designed to describe the number of consultations with an ED unit in Japan before and during the COVID-19 pandemic. It is important to follow the trends seen at ED units over time to further understand the pathogenesis of the disorders and provide useful ED support under special environmental conditions.

### Aim

This study aimed to examine the effect of COVID-19 on the number of cases reported to the Chiba Prefecture Eating Disorder Treatment and Support Center (CSC), including for age- and disorder-specific subgroups.

## Methods

### Japanese school life and the status of the COVID-19 pandemic in Japan

In Japan, the school year for junior and senior high school students begins in April. Students and employees often change positions at this time of the year. Summer holidays begin in late July, and the second school term starts in September. The following March is the graduation ceremony, and businesses commence operations in April. March to April thus represents the transfer season.

The number of new COVID-19 cases in Japan each month was investigated based on public information from the government. The following data were gathered to examine the contextual factors: the first case in Japan, the declaration of a domestic emergency, and the request for the temporary closure of all schools nationwide.

### Relationship between COVID-19 and the number of consultations by age

**Fig. 1:**
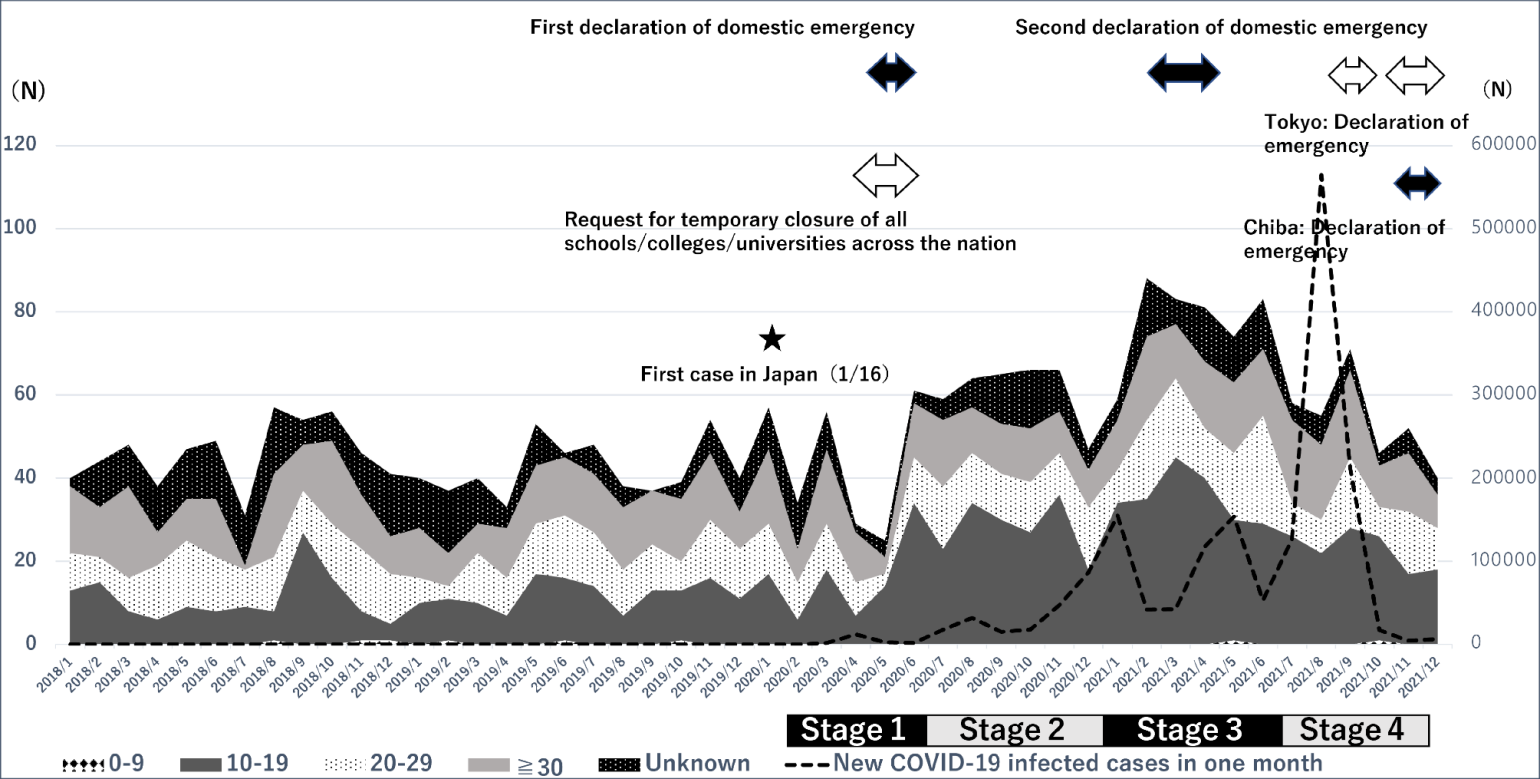
Overview of number of consultations by age group, COVID-19 pandemic stage, and government agency response *Note*: The left vertical axis denotes the number of consultations per month. The right vertical axis denotes the number of new COVID-19 infections per month. Both arrows indicate the duration of living restrictions. COVID-19 coronavirus disease 2019

On January 16, 2020, Japan confirmed its first COVID-19 case. The government subsequently requested the temporary closure of educational institutions from March 2 to May 31, 2020; most schools complied (see Fig. 1 for a timeline).

COVID-19 lockdown requirements vary globally, from curfews with penalties to curfew requests [11]. In Japan, requests to limit non-essential outings, educational institution closures, restrictions on restaurant activities, and staggered work hours were implemented via a declared domestic emergency, without using lockdown terminology.

Japan’s first state of emergency was in place from April 7 to May 25, 2020. During this period, some facilities offered online classes. The second state of emergency was in place from January 8 to March 21, 2021. Subsequently, states of emergency were no longer declared nationally; instead, it was issued at the prefecture level, depending on the infection situation. A state of emergency in Chiba Prefecture was in place from August 2 to September 30, 2021.

The peak monthly number of new COVID-19 cases during the study period (January 2020–December 2021) was 210,672 in September 2021. The total number of COVID-19 infections in Japan during the study period was 1,729,578, of which there were 18,395 total deaths. The number of COVID-19 infections in the United States during this period was 54,269,566, and with 824,580 deaths.

### Study design

We recorded the number and types of consultations provided by the CSC coordinator. The study period extended from January 2018 to December 2021, which included the two years before and after the onset of the COVID-19 pandemic.

Centers like the CSC provide medical care information for patients and their families, educate therapists and train doctors from other medical institutions, and promote medical cooperation. The National Center of Neurology and Psychiatry coordinates the National Core Center for Eating Disorders, including our CSC [12]. The authors have been conducting the aforementioned activities, such as the consultation services and promoting medical cooperation at the CSC since 2017. A 2020 survey revealed that Chiba Prefecture has a well-balanced metropolitan–rural population distribution of 6.27 million people [12]. It is the sixth most densely populated region in Japan. Chiba Prefecture also boasts the twelfth-highest per capita income, the tenth-highest total number of farms, and the ninth-highest number of hospitals. Therefore, we considered the activity of the CSC to be a useful metropolitan–rural model of an eating disorder support center in Japan.

### Inclusion and exclusion criteria

Participants comprised patients with EDs who were currently visiting a hospital (clinical cases in treatment), those who had previously visited the hospital but had stopped treatment despite the presence of ED symptoms (clinical cases who had dropped out of treatment), and subclinical cases of individuals with ED symptoms who had never visited a hospital for their ED symptoms (subclinical cases). For patients who were attending a hospital/clinic or interrupted medical treatment due to various reasons, as we mention in the introduction, EDs were diagnosed by a medical doctor. The criteria for diagnosis were in accordance with the Diagnostic and Statistical Manual of Mental Disorders, Fifth Edition [13] or the International Statistical Classification of Diseases and Related Health Problems, 10th Revision [14]. Patients who did not visit a hospital were considered “subclinical cases” based on the following information provided by the coordinator: (i) those who exhibited a strong preoccupation with their weight or body shape or abnormal eating behaviors, but with no obvious organic diseases, such as malignancy or gastrointestinal disease, that would cause them to rapidly gain or lose weight, and (ii) those with a body mass index (BMI) of less than 18.5 and in denial about the seriousness of the their physical condition. Other sources included family members, school personnel, and administrative agencies. In cases where family members, schools, hospitals, or health centers were consulted, the classification was based on the age of the individual with the ED, not the age of the family member.

**Fig. 2:**
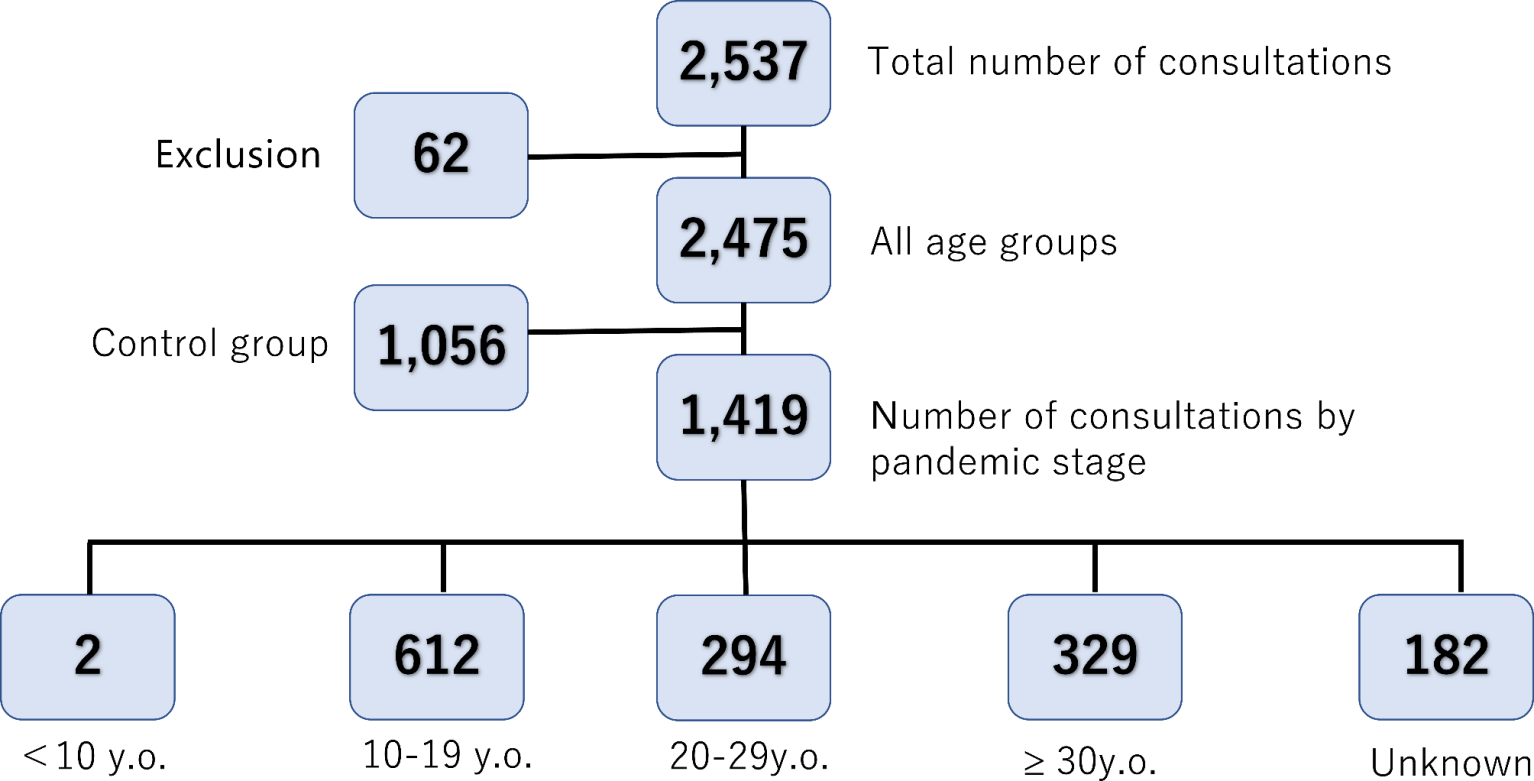
Number of consultations by age group and COVID-19 pandemic stage.*Note*: <10 y.o.: 0 to 9 years old; 10–19 y.o.: 10 to 19 years old. 20–29 y.o.: 20 to 29 years old; ≥30 y.o.: Over 30 years old. Unknown: Age unknown. *COVID-19* coronavirus disease 2019

The total number of consultations was 2,537, of which the data of 2,475 were available for analysis. Of these cases, 1,901 (76.8%) completed one consultation (Fig. 2). Consultations for older patients with dysphagia, but without an ED, were excluded (62 cases). In other cases, consultations were conducted more than once, with the content of the second and subsequent consultations often differing and with a gap between consultations; thus, multiple consultations for a single case were counted as different cases. There remains a risk that significant differences are too often found when using this method. Patients aged 10 years or younger were excluded from the subgroup analyses because of the low number of eligible participants in this age range. The timeline was categorized as follows: Stage 1: January 2020–June 2020 (including the first domestic state of emergency); Stage 2: July 2020–December 2020; Stage 3: January 2021–June 2021; and Stage 4: July 2021–December 2021 (Fig. 1).

### Data sources and procedure

All telephonic or e-mail consultations and support were handled by trained nurses and clinical psychologists (coordinators). They were regularly supervised by a physician who specializes in treating EDs. The coordinators from Japan’s four ED treatment and support centers convene for training once yearly. Using the platform shared by Eating disorder support centers in Japan, the following details were compiled: demographic characteristics, including sex, age, diagnosis of ED, body weight (a BMI of less than 18.5, a BMI of over 18.5, unknown), and location; as well as the contents of the consultation, including current status (no medical examination, in treatment/dropped out of treatment, unknown), educational background, the content of the consultation, details of eating behavior abnormalities, mental status (e.g., anxious, depressed), and presence of self-harming behavior. Among these data, only age, BMI, current treatment status, and the source of the data were extracted (Table 1). For telephone calls, a clinical interview was conducted, and additional comments were noted in the free-text field. E-mails were sent to the consultant about their participant’s problems such as guidance on where to receive specialist medical care and knowledge about, for example, the disease and how to cope with the disease or and how well these issues had been resolved.

**Table 1:** Number of consultations by pandemic stage

	Before pandemic Jan 2018 to Dec 2019	After pandemic Jan 2020 to Dec 2021	*Stage 1	Stage 2	Stage 3	Stage 4
All age groups	1056	1419	262	367	468	322
Under 10 years	6	2	0	0	1	1
10 to 19 years	271	612	96	168	212	136
20 to 29 years	262	294	54	75	100	65
Over 30 years	305	329	73	71	94	91
Unknown	212	182	39	53	61	29
BMI 18<consultations	446	864	164	225	280	195
BMI 18 ≧ consultations	163	266	43	75	91	57
Unknown	447	289	55	67	97	70
Consultation from patient	322	467	80	135	147	105
Consultation from family members	588	825	159	201	282	183
Consultation from school/hospital/university	54	61	12	19	18	12
Other or unknown	92	66	11	12	21	22
Subclinical case	332	511	85	160	160	106
Clinical case (in treatment)	277	590	116	138	205	131
Clinical case (dropped out of treatment)	166	212	41	42	72	57
Unknown	281	106	20	27	31	28

* Post-pandemic period was divided into six-month intervals. The number of people is described separately for Stages 1–4

*BMI* Body mass index

### Statistical analysis

To evaluate the impact of the pandemic on the number of consultations, the Seasonal Decomposition of Time Series by Loess (STL decomposition) [15] was applied to the time series data of monthly consultations to decompose them into trend components, seasonal components, and residuals. In addition, the period of the seasonal component was set as 12. The STL decomposition was carried out using the STL function of the stats package in R version 4.3.0. The study was approved by the Ethics Committee of the National Center for Global Medical Research (NCGM-G-003196-00).

## Results

### Basic data

The data of 1,419 enrolled participants (after the COVID-19 onset) were available for analysis. In this group, the mean age of those with known age was 24.0 ± 11.4 years (N = 1228), while the age of the remaining 191 participants was unknown. The control group (before COVID-19 onset) included 1,056 participants. In the control group, the mean age of those with a known age was 27.3 ± 12.51 years (N = 837). The remaining 219 participants were of unknown age. The mean ages of the intervention and control groups differed significantly (*p* < 0.0001). The intervention group comprised 58 males and 1,294 female participants, and the sex of 67 individuals was unknown. The control group comprised 45 males, 927 females, and 84 participants of unknown sex. The difference between male and female participants before and after the onset of the disease was not significant (*p* = 0.157). The ratio of subclinical cases who did not visit a hospital was 25.2%. As shown in Fig. 1, two cases were aged under 10 years, 612 were aged 10–19 years (comprising most respondents), 294 were aged 20–29 years, 329 were aged 30 years and above, and 182 did not report their age at the time of the survey (unknown).

### Overview of the trends in the number of consultations by age group (Fig. 1)

The highest number of total consultations was recorded in March 2021, approximately six months before the peak number of new cases of COVID-19. The increase in the number of consultations tended to subside marginally throughout the year following the onset of the pandemic.

According to the STL decomposition, original data = seasonal components + trend components + residuals. The total number of consultations increased after the onset of the pandemic and peaked around the beginning of 2022, before falling back down again. Among the sub-analyzed data, participants in their teens, twenties, with a BMI < 18.5, in treatment, individual, family member, clinical case in treatment, and subclinical case exhibited similar trends.

**Fig. 3:**
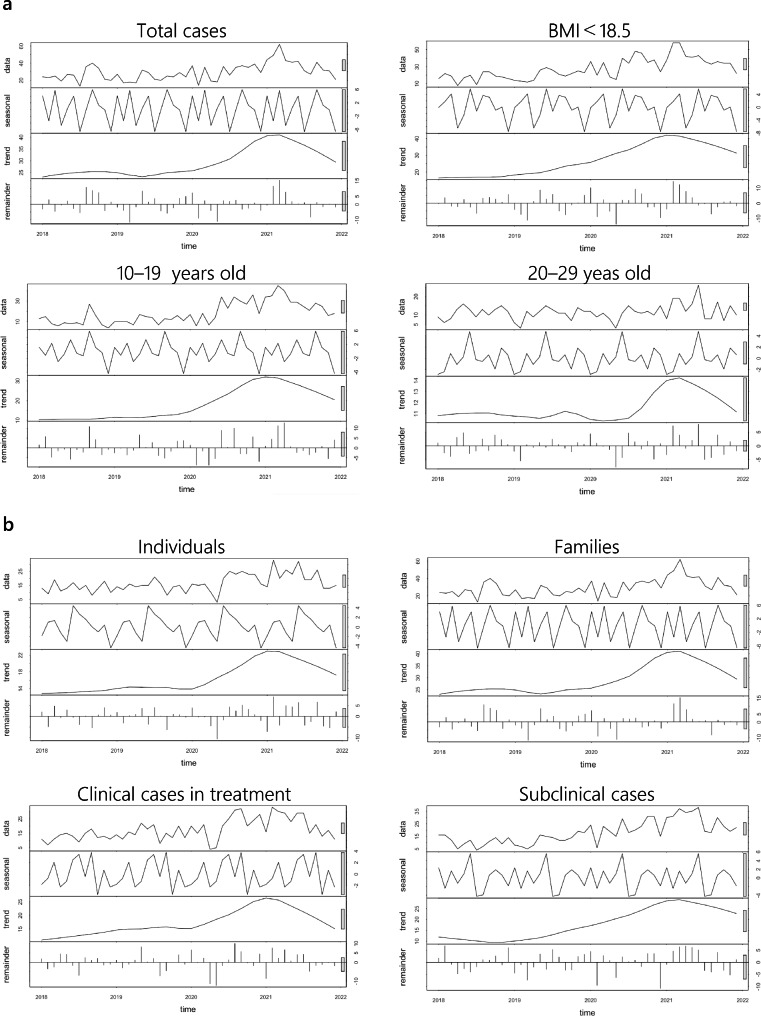
**a** STL results for total cases, BMI subgroups, and 10–19- and 20–29-year age groups. *Note*: The horizontal axis indicates time. The vertical axis indicates data, seasonal components, trend components, and residuals. *BMI* Body mass index, *STL* Seasonal Decomposition of Time Series by Loess. **b** STL results for individuals, families, clinical cases in treatment, and subclinical cases. *Note*: The horizontal axis indicates time. The vertical axis indicates data, seasonal components, trend components, and residuals. *STL* Seasonal Decomposition of Time Series by Loess

For individuals under 10 years old and with consultations extended by schools, hospitals, and administrative agencies such as health centers, statistical analyses were not performed because of the low consultation numbers. In addition, to exclude small groups, factors with a trend scale of 10 or less were eliminated from the STL decomposition (Fig. 3).

## Discussion

Our findings indicate that the number of consultations transiently increased during the early stages of the COVID-19 pandemic, a result supported by our sub-analysis. This phenomenon might be associated with environmental factors, such as the restart of school life and interactions with others. During the pandemic, pediatric ED admissions showed an average increase of 83% compared to 16% for adults [4]. Similarly, Takakura et al. highlighted that patients with EDs tended to be younger in the period immediately following the onset of COVID-19 compared to previous years [9]. Kurisu et al. reported an increase in patients with EDs up to March 2021 [10]. The increase in the number of consultations in the 10–20 ages group in our study supports these results. Our study has a longer follow-up than the above-mentioned studies, which is why there was a decrease in the number of consultations at least one year after the start of the pandemic.

Our results are comparable to those of previous studies. Furthermore, this study included individuals who did not visit a hospital. Diagnostic uncertainty is inevitable. However, a phone consultation may be easier than an in-hospital consultation for a patient with low motivation. In addition, internalized and treatment-related stigma in patients with EDs is most frequently associated with reduced help-seeking behavior [16]. Nonetheless, the role of the internet and online resources as an aid for offline help-seeking must be considered for EDs [16]. Therefore, telephone consultations, which are easier for patients to access than medical examinations, may be useful. We believe that this survey contributes to the literature and has strong social implications because it identifies many potential patients with EDs who do not visit hospitals or seek treatment.

Regarding the increasing number of those aged 10–20 seeking consultations, previous studies have shown that EDs in children and adolescents are more susceptible to deterioration due to changes in their home and school environments, such as lockdowns and school closures [17]. The following theories were formulated to explain the increase in hospitalized patients with anorexia nervosa during the pandemic: “Disruption of eating and exercise habits”; “a combination of social isolation and school/college/university closures has disconnected patients from protective factors”; and “the reduction in extracurricular activities, school routine, and peer relationships has created room for eating disorder cognitions to intensify without the usual distractions” [3, 17]. Social isolation and disruption of social networks due to COVID-19 lockdowns increased the risk of relapse in individuals with EDs and those with psychiatric disorders [18].

In this study, it was impossible to conclude with total confidence the exact factors that led to an increase in the number of consultations of adolescent patients with EDs due to the COVID-19 pandemic. However, before vaccination drives were sufficiently widespread in Japan, social distancing and staying at home were important policies. Stages 1–3 of this study occurred under these circumstances. Thus, the following theories were formulated: (i) forced and prolonged home confinement increased the stressors on children at home [19]; (ii) there was a sense of loss of control at home due to the uncertainty of the future [19]; (iii) people could not access treatment, which increased the need for consultations [18]; and (iv) family members of individuals with EDs may have become more aware of the problems because they were spending more time together [9, 20]. The average age of patients with EDs was younger [20] after the pandemic than before it.

The number of patient consultations declined unexpectedly in the second half of Stage 4, when the state of emergency was still ongoing, implying that the impact of the COVID-19 pandemic on EDs and subclinical cases was transient. Based on previous studies [17, 18] and considering the variation in the number of consultations over time, including the national circumstances, we posit the following: Stage 1 was affected by sudden changes in the environment, such as the sudden and temporary closure of all schools nationwide, but this stressor may have been gradually reduced due to changes in government policy; Stage 2 was marked by the emergence of stressors within the family; and in Stage 3, once schools resumed operations, stress was visibly reduced in children and adolescent patients. Unlike adults, who were stressed by social distancing, those aged 10–20 may have had means of communication with one another, such as mobile phones and social networking service (SNS), which they were likely to have used when they were unable to interact directly with friends. Although the number of consultations per month has decreased, the number of patients has increased, not to mention the importance of post-onset treatment. The decrease in opportunities to examine communication skills in groups, such as school and club activities, may have provided a temporarily less stressful environment for patients with EDs. It is unclear whether the persistent avoidance of this reality (no group activities in schools or clubs) has a genuinely positive effect on the patient’s subsequent course of treatment. For some patients, the COVID-19 pandemic may have had a positive effect [4].

A clinical implication of this study is that it reveals that the COVID-19 pandemic affects not only patients with diagnosed EDs but also cases that have not visited the hospital. This aspect was mainly observed in adolescents. Another clinical significance is that the increase in the number of consultations was transitory. The significance of this is unclear because no qualitative study was conducted; however, teenage patients who are good at social networking may have used it to resume communication, even if their daily routines were restricted.

EDs are intractable [21]; therefore, amid the transient increase in this study, anorexia nervosa should not be taken lightly. In addition, the results of this study have advanced our understanding of the pathophysiology of EDs. We recognize the importance of maintaining social distance to protect ourselves and others from COVID-19 while also acknowledging the importance of reestablishing social life in group settings for patients with EDs. Infectious disease researchers predict that the COVID-19 pandemic will likely persist for several years, necessitating a continued vigilant response.

### Limitations

The data used in this study may be biased because the study was conducted with single-center data. Because the data were not obtained from hospital medical records but from telephone and e-mail consultations, the subclinical cases reflect the judgement of the coordinator and not a definitive medical diagnosis, and the presence or absence of post-traumatic stress disorder (PTSD) and other comorbid psychiatric disorders may have been inadequately assessed. Our study cannot examine the presence or absence of comorbid psychiatric disorders. It has been reported that individuals experiencing EDs with comorbid depression, anxiety, and PTSD are more susceptible to lockdowns imposed by the COVID-19 pandemic [4, 22]. Detailed evaluation of the participants and the content of consultations remains an issue to be analyzed in future studies.

## Conclusions

The COVID-19 pandemic transiently increased consultation numbers. This trend was supported by subclinical analyses, such as adolescent ED cases and individual clinical and subclinical cases not involving hospital visits. An inference can be made that EDs are significantly affected by environmental changes, such as educational institution closures. Although the COVID-19 pandemic persists, its impact on EDs was transient once the social isolation factors were removed.

## Data Availability

The dataset generated during the current study is not publicly available but is available from the corresponding author upon reasonable request.

## Declarations

**Ethics approval and consent to participate**.

This study was approved by the Ethics Committee of the National Center for Global Medical Research (NCGM-G-003196-00). Consent was obtained in the form of an opt-out option.

**Consent for publication**.

## Abbreviations

BMI: body mass index
COVID-19: coronavirus disease 2019
CSC: Chiba Prefecture Eating Disorder Treatment and Support Center
ED: eating disorder
PTSD: post-traumatic stress disorder
SARS-CoV-2: severe acute respiratory syndrome coronavirus 2
STL: Seasonal Decomposition of Time Series by Loess
